# Long noncoding RNA DLX6-AS1 promotes neuroblastoma progression by regulating miR-107/BDNF pathway

**DOI:** 10.1186/s12935-019-0968-x

**Published:** 2019-11-27

**Authors:** Huan-yu Zhang, Mao-qing Xing, Jing Guo, Jin-chuan Zhao, Xin Chen, Zhong Jiang, Hong Zhang, Qian Dong

**Affiliations:** 1 Department of Pediatric Surgery, The Affiliated Hospital of Qingdao University, Jiangsu Road 16, Qingdao, 266000 Shandong China; 2 Shandong Key Laboratory of Digital Medicine and Computer Assisted Surgery, The Affiliated Hospital of Qingdao University, Jiangsu Road 16, Qingdao, 266000 Shandong China

**Keywords:** Neuroblastoma, lncRNA DLX6-AS1, Proliferation, Invasion and migration, miR-107, Brain‐derived neurotrophic factor

## Abstract

**Background:**

Long noncoding RNAs (lncRNAs) play essential roles in tumor progression. However, the functions and targets of lncRNAs in neuroblastoma (NB) progression still remain to be determined. In this study, we aimed to investigate the effect of lncRNA DLX6 antisense RNA 1 (DLX6-AS1) on NB and the underlying mechanism involved.

**Methods:**

Through mining of public microarray datasets, we identify aberrantly expressed lncRNAs in NB. The gene expression levels were determined by quantitative real-time PCR, and protein expression levels were determined by western blot assay. 3-(4,5-dimethylthiazol-2-yl)-2,5-diphenyltetrazolium bromide (MTT) assay, colony formation assay, wound-healing assay, transwell invasion assays and flow cytometry analysis were utilized to examine cell proliferation, migration, invasion and apoptosis. Luciferase reporter assay was performed to confirm the interaction between DLX6-AS1and its potential targets. Tumor xenograft assay was used to verify the role of DLX6-AS1 in NB in vivo.

**Results:**

We identified DLX6-AS1 was upregulated in NB by using a public microarray dataset. The expression of DLX6-AS1 was increased in NB tissues and derived cell lines, and high expression of DLX6-AS1 was positively correlated with advanced TNM stage and poor differentiation. Knockdown of DLX6-AS1 induced neuronal differentiation, apoptosis and inhibited the growth, invasion, and metastasis of NB cells in vitro and impaired tumor growth in vivo. MiR-107 was the downstream target of DLX6-AS1. MiR-107 was found to target brain‐derived neurotrophic factor (BDNF) which is an oncogene in NB. Knockdown of miR-107 or overexpression of BDNF reversed the suppression of NB progression caused by DLX6-AS1 silence.

**Conclusion:**

Overall, our finding supports that DLX6-AS1 promotes NB progression by regulating miR-107/BDNF pathway, acting as a novel therapeutic target for NB.

## Background

Neuroblastoma (NB) is the most common solid extracranial malignancy of childhood and accounts for 15% of all childhood cancer deaths [1]. The biological behavior of NB is extensively heterogeneous, ranging from spontaneous regression to rapid progression [2]. For high-risk NB patients, many therapeutic modalities fail to improve the clinical outcome [2]. Better elucidating the mechanisms for the aggressive progression of NB is needed for improving the therapeutic efficiencies.

Long noncoding RNAs (lncRNAs) are a newly discovered class of noncoding RNAs (ncRNA) that are longer than 200 nucleotides and are not translated into proteins [3]. Increasing researches have illustrated that lncRNAs are involved in multiple biological processes including cell proliferation, cell cycling, apoptosis, and metastasis. Recent evidence has shown the emerging roles of lncRNAs in the pathogenesis of NB [4]. For example, Ets-1 promoter-associated noncoding RNA (pancEts-1) is overexpressed in NB, and promotes neuroblastoma progression through hnRNPK-mediated β-catenin stabilization [5]. Loss of neuroblastoma-associated transcript-1 (NBAT-1) contributes to aggressive NB by increasing cellular proliferation, invasion and impairing differentiation of neuronal precursors [6]. MicroRNAs (miRNAs) is another type of ncRNA with 18–22 nucleotides length [7]. Dysregulation of certain miRNAs has been implicated in the pathogenesis of NB [7]. For example, miR-181a/b is significantly upregulated in aggressive neuroblastoma, which enhanced its tumorigenesis and progression by suppressing the expression of ABI1 [8]. Our previous studies show that miR-145 inhibits the NB progression through targeting hypoxia-inducible factor 2 alpha (HIF-2α) [9]. Recently, accumulating articles revealed that one potential function of lncRNAs was to directly interact with miRNAs as a sponge and regulate their expression and activity [10]. For example, LINC00152 promotes cell growth and invasion of papillary thyroid carcinoma by regulating the miR‐497/BDNF axis [11]. However, the functions of lncRNA on miRNAs in the pathogenesis of NB has not been described. Thus, it is currently necessary to further investigate the roles of lncRNAs in NB progression.

In the present study, through mining of a public microarray dataset, we identified DLX6-AS1 associated with poor outcome of NB. We demonstrate that DLX6-AS1 is up-regulated in collected NB tissues and cell lines. Moreover, in vivo and in vitro assay showed that knock down of DLX6-AS1 induced neuronal differentiation, apoptosis and inhibited the proliferation, migration, invasion and tumor growth of NB cells. Our results and findings reveal the potential insight of DLX6-AS1/miR-107/BDNF pathway during NB tumorigenesis.

## Materials and methods

### Patient tissue samples

Our collected samples and clinical data were obtained with the informed consent of all legal guardians of the patients. Approval to conduct this study was obtained from the Ethics Committee of the Affiliated Hospital of Qingdao University. Human normal dorsal root ganglia were collected from interrupted pregnancies. Fresh tumor specimens of a total of 36 NB patients were collected at surgery and stored at − 80 °C until use. Total RNAs of normal human dorsal ganglia pooling from 18 male/female Caucasians were obtained from Clontech (Mountain View, CA, USA).

### Data mining of public database

The Gene Expression Omnibus (GEO) database (https://www.ncbi.nlm.nih.gov/geo/) was used to retrieve the relevant datasets. The differentially expressed lncRNAs between different groups were screened by unpaired Student’s t test and correction multiple testing method. Log-rank test and correction multiple testing analyses were applied to evaluate the survival significance of each lncRNA in NB patients.

### Cell culture

Human NB cell lines NB-1643, NB-1691, SK-N-AS, IMR-32, SH-SY5Y and SK-N-SH were obtained from American Type Culture Collection (ATCC, Rockville, MD). Cells were grown in RPMI1640 medium (Life Technologies, USA) containing 10% fetal bovine serum (Gibco Termo Fisher Scientifc, USA), penicillin (100 U/mL), and streptomycin (100 mg/mL) in a humidified incubator at 37 °C with 5% CO_2_.

### Gene over-expression and knockdown

Lentivirus expressing shDLX6-AS1 or shRNA control were designed and packaged by Genechem (Shanghai, China). Stable cell lines were established by infecting lentivirus into SK-N-SH cells and selected by puromycin (Sigma, St. Louis, USA). The small interfering RNAs (siRNAs) for DLX6-AS1 (siDLX6-AS) and the negative control siRNA, miR-107 mimics, miR-107 inhibitors and the negative control miRNAs were purchased from RiboBio (Guangzhou, China). Human BDNF overexpression plasmid (pcDNA-BDNF) and blank plasmid (pcDNA3.1) were obtained from GenePharma (Shanghai, China). The transiently transfection for Small interfering RNA or pcDNA plasmid was conducted using Lipofectamine 2000 Transfection Reagent (15596‐026; Invitrogen, Carlsbad, CA) according to the manufacturer’s instructions and the transfection efficiency was confirmed using quantitative real-time PCR (qRT-PCR). Small interfering RNAs contain si-DLX6-AS1, 5′-AAUAAAGAACACUUACACUACUG-3′; miR-107 mimics, 5′-AGCAGCAUUGUACAGGGCUAUCAAUAGCCCUGUACAAUGCUGCUUU-3′; miR-107 inhibitors,5′-UGAUAGCCCUGUACAAUGCUGCU-3′. Each experiment was performed in triplicate.

### Quantitative real time polymerase chain reaction (qRT-PCR)

Total RNA isolation from NB cells and frozen tissue was performed using TRIzol reagent (Invitrogen). Then, cDNA was constructed from total RNA using the PrimeScript first Strand cDNA Synthesis Kit (6110A; TaKaRa, Dalian, China). Real-time PCR was carried out using One Step SYBR RT‐PCR Kit (RR086A; TaKaRa) and measured by ABI 7900 system. The following primers were used to detect the expression of DLX6-AS, miR-107, BDNF, and internal control U6 and GAPDH: DLX6-AS1, forward: 5′-CCAAATGCTACCATCCAGCC-3′, reverse: 5′-TCTGGCTTCCCTTAACCAAAA-3′; miR-107, RT, 5′-GTCGTATCCAGTGCAGGGTCCGAGGTATTCGCACTGGATACGACTGATAG-3′, forward: 5′- AGCAGCATTGTACAGGG-3′, reverse: 5′- GTGCAGGGTCCGAGGT-3′; BDNF, forward: 5′-TCCCTGGCTGACACTTTT-3′, reverse: 5′-ATTGGGTAGTTCGGCATT-3′; U6, RT, 5′-AACGCTTCACGAATTTGCGT-3′, forward: 5′-CTCGCTTCGGCAGCACA-3′, reverse: 5′-AACGCTTCACGAATTTGCGT-3′; GAPDH, forward, 5′-CGACACTTTATCATGGCTA-3′, reverse, 5′-TTGTTGCCGATCACTGAAT-3′. The transcript levels were analyzed by 2^−∆∆Ct^ method. Each experiment was performed in triplicate.

### Proliferation assays

Cell proliferation status was determined with the 3‐(4,5‐dimethyl‐2‐thiazolyl)‐2,5‐diphenyl‐2‐*H*‐tetrazolium bromide (MTT) assay and colony formation assay. For MTT assay, the transfected cells were seeded in 96-well plates at the density of 1 × 10^3^ per well and then cultured for 1, 2, 3, 4 and 5 days. Then, 20 μL of MTT (5 mg/mL) (Roche, Basel, Switzerland) solution was added and incubated at 37 °C for 2 h. The optical density (OD) values were read at 490 nm by a microplate reader (Molecular Devices, LLC, Sunnyvale, CA, USA). Percent viability was defined as the relative absorbance of intervened cells versus control cells. For colony formation assay, the transfected cells were seeded in 6-well plates at the density of 500 per well for 2 weeks. After 14 days, colonies were fixed with 4% paraformaldehyde for 5 min and stained with 0.1% crystal violet for 10 min. Each experiment was performed in triplicate.

### Wound healing assay

Transfected cells were seeded in six‐well plates, and grown to 100% confluency. The confluent monolayer of cells was scratched with a plastic apparatus to create a clear cell‐free zone. Subsequently, the cells were incubated in serum-free medium and cultured for 24 h. Wound closure was measured using Nikon NIS‐Element Basic Research v3.2 software. The migration assays were performed independently in triplicate.

### Cell invasion assays

The invasion assay was performed using Transwell chambers with Matrigel (8 µm pore size, BD Biosciences, Franklin Lakes, NJ, CA, USA). At 48 h after transfection, 1 × 10^5^ NB cells were resuspended in 200 μL serum-free medium and placed on upper chambers and 600 μL medium containing 10% FBS was added into lower chambers. After 24 h of incubation, cells were fixed with polyoxymethylene. Finally, the invaded cells were counted from five randomly fields under a microscope. Each experiment was performed in triplicate.

### Flow cytometry analysis

Apoptosis ratios of cells were determined by Annexin V/FITC and PI staining flow cytometry. Briefly, the transfected NB cells were harvested, digested and suspended in 100 μL binding buffer. Then the cells were stained with 10 μL Annexin V/FITC (BD Pharmingen, San Diego, CA) for 10 min and 5 μL PI (20 mg/mL) for 5 min on ice in the dark at room temperature. The apoptotic cells were analyzed by a flow cytometer (BD Biosciences, San Jose, CA, USA) at a wavelength of 488 nm.

### Western blot

Proteins were extracted from tissues using RIPA lysis buffer (Roche, Diagnostics, Mannheim, Germany). Then, the lysate was loaded into SDS-PAGE, and transferred to PVDF membranes (Millipore, USA). After blocking with 5% milk at room temperature, the membranes were incubated with primary antibodies against BDNF (ab220679, 1:1000; Abcam Inc., Cambridge, UK), GAP43 (ab11136, 1:1000; Abcam Inc.), NF-200 (ab8135, 1:1000; Abcam Inc.) or GAPDH (ab9485, 1:3000; Abcam Inc.) at 4 °C overnight. Subsequently, the membranes were incubated in HRP‐conjugated secondary antibody, goat antirabbit to IgG (ab205718, 1: 20,000; Abcam Inc.) for 1 h at room temperature. The expression was tested with enhanced chemiluminescence (ECL, Millipore, Bredford, USA) and ImageJ (NIH, USA) was used for image densitometric analysis and GAPDH was used as the internal control. Each experiment was performed in triplicate.

### Luciferase reporter assays

Fragments of DLX6-AS1 and BDNF 3′-UTR containing miR-107 binding sites (WT) were amplified by RT-PCR and cloned into the luciferase report vector pGL3-basic (Promega, Madison, WI., USA). Point mutations were generated in the miR-107 binding site of the DLX6-AS1 fragment and BDNF 3′-UTR using the Site-Directed Mutagenesis kit (Invitrogen, Carlsbad, CA, USA). HEK293T cells were co-transfected with luciferase plasmids and miR-107 mimics, or mimics NC by using Lipofectamine 2000 reagent according to the manufacturer’s instruction (Invitrogen). The luciferase activity of Firefly luciferase compared to Renilla luciferase was measured 24 h after transfection according to the manufacturer’s protocol (Promega). Each experiment was performed in triplicate.

### In vivo tumorigenesis

All animal experiments were carried out in accordance with NIH Guidelines for the Care and Use of Laboratory Animals, and approved by the Animal Care Committee of the Affiliated Hospital of Qingdao University. Male BALB/c athymic nude mice at 4–6 weeks were purchased from Shanghai SLAC Laboratory Animal Co. All mice were maintained in a barrier facility at Animal Center of Qingdao University. For tumor formation assay, 1 × 10^6^ SK-N-SH cells stably expressing shDLX6-AS1 or shNC were subcutaneously injected into the right flank of mice (n = 5 per group). Tumor size was measured every 4 days using (length × width^2^) × 0.5. Tumor weight was measured after sacrifice of mice. For survival analysis, mice were subcutaneously inoculated with 5 × 10^6^ stable transfected SK-N-SH cells (n = 12 per group), and survival time of the mice were monitored at the end of observation.

### Statistical analysis

All Data were shown as mean ± standard deviation (SD). The cutoff values were determined by average gene expression levels. The data analysis was performed by using GraphPad Prism (Version 7.0, GraphPad, San Diego, USA). Correlations between expression and clinicopathological characteristics were analyzed by Chi-square test. Student’s t test or one-way ANOVA were used for intra-group comparison, as appropriate. Kaplan–Meier’s method and the log-rank test were performed for survival rate p value < 0.05 was considered statistically significant.

## Results

### DLX6-AS1 expression is upregulated in NB

**Fig. 1:**
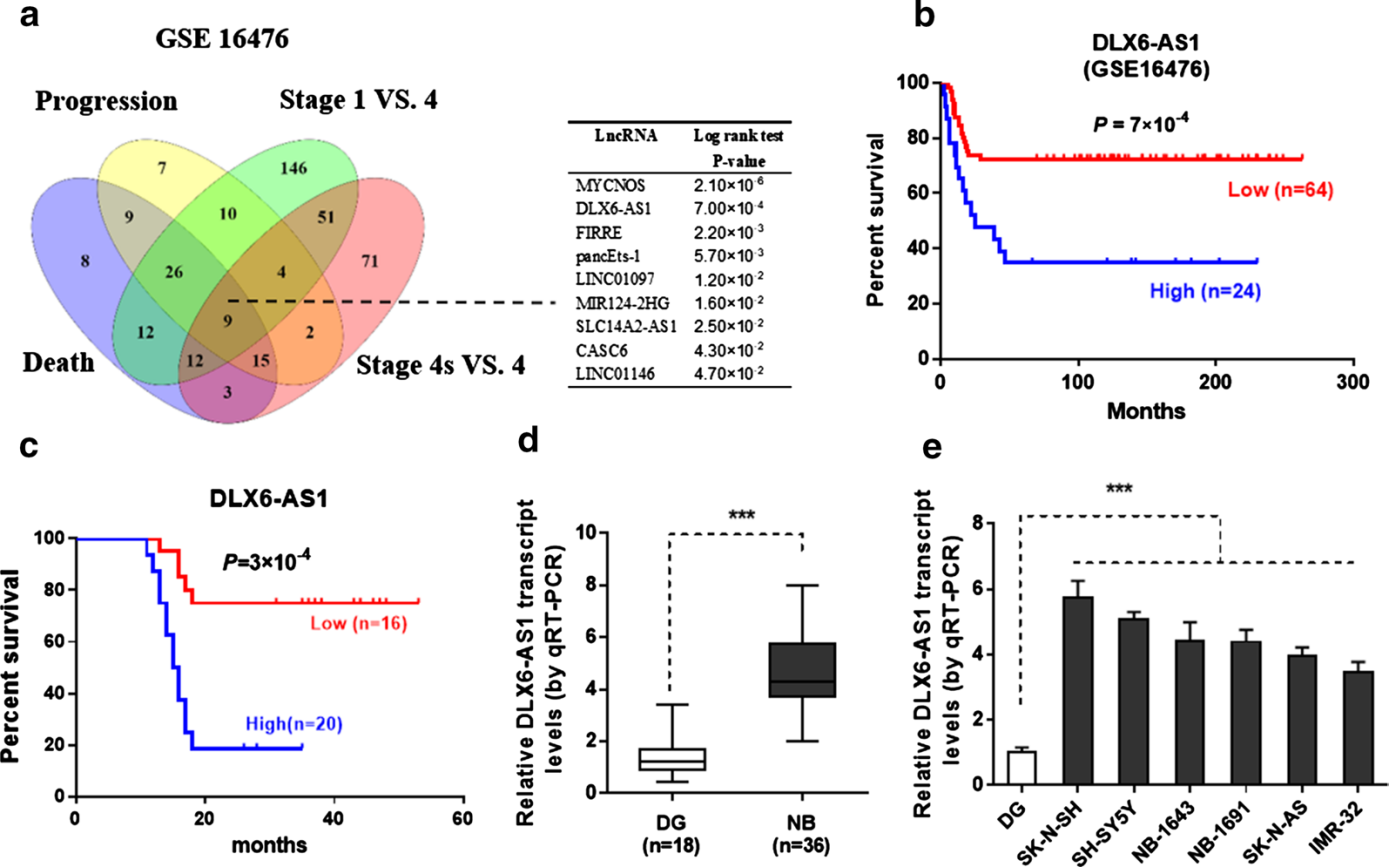
DLX6-AS1 expression is upregulated in NB tissues and cell lines. **a** Venn diagram (left panel) and Log-rank test (right panel) indicated the identification of differentially expressed lncRNAs (fold change > 1.3, *p *< 0.05) in 88 NB cases (GSE16476) with various status of death, progression, and INSS stages, and their association with outcome of patients. Kaplan–Meier curves showed overall survival with high or low DLX6-AS1 expression in **b** public data of 88 (GSE16476) patients and in **c** our collected data of 36 NB patients. **d** The qRT-PCR assay indicated the DLX6-AS1 expression (normalized to GAPDH) in NB tissues (n = 36) compared with normal dorsal ganglia (DG, pooling n = 18). **e** The qRT-PCR assay showed the DLX6-AS1 expression (normalized to GAPDH) in normal dorsal ganglia (DG, pooling n = 18) and NB cell lines. ***p < 0.001

Initially, microarray dataset (GSE16476) [12] of 88 NB patients was acquired from GEO. We identified 93, 82, 271, and 167 differentially expressed (fold change > 1.3, p < 0.05) lncRNAs in NB tissues with varied status of death, clinical progression, and international neuroblastoma staging system (INSS) stages (stage 1 versus 4 and stage 4S versus 4), respectively (Fig. 1a). To further search for the differentially expressed genes in NB, 9 lncRNAs were found to be consistently associated with the above clinical parameters via over-lapping analysis of these lncRNAs (p < 0.001) (Fig. 1a). Log-rank test analysis revealed top four lncRNA predicting prognosis of NB patients (Fig. 1a). Among them, the functions of lncRNAs pancEts-1 [5] and MYCNOS [13] in NB have been reported, while the roles of DLX6-AS1 in the tumorigenesis and aggressiveness of NB still remain elusive. The expression of DLX6-AS1 in NB tissues was further divided into low expression group and high expression group based on the median values. Kaplan–Meier curves of these 88 NB cases (GSE16476) showed significant difference in overall survival (*p *= 5 × 10^−3^) between the high and low DLX6-AS1 expression groups (Fig. 1b), which was accordant with our collected samples with 36 NB patients (Fig. 1c). We validated that the expression of DLX6-AS1 was significantly increased in an independent cohort of 36 primary NB tumors compared to those in normal dorsal ganglia (Fig. 1d). High expression of DLX6-AS1 was positively correlated with poor differentiation or advanced TNM stage (Table 1). We then performed qRT-PCR to analyze DLX6-AS1 expression in NB cell lines. As compared with normal dorsal ganglia, higher levels of DLX6-AS1 expression was seen in all NB cell lines (Fig. 1e). To explore the roles of DLX6-AS1 in NB progression, NB cell lines representing higher expression levels were applied. These observations suggested that DLX6-AS1 might be involved in the regulation of NB development.

**Table 1:** Correlation between DLX6-AS1 expression and clinical characteristics

Variable	DLX6-AS1 expression		*p* value
	Low (n = 16)	High (n = 20)	
Age			
< 2.5	9	12	0.723
≥ 2.5	7	8	
Gender			
Male	10	11	0.662
Female	6	9	
Lymph node metastasis			
Yes	5	14	0.038
No	11	6	
INSS stage			
1–2	7	5	0.025
3–4S	9	15	
Differentiation			
Well	10	5	0.019
Moderate-poor	6	15	

*p* < 0.05 was considered statistically significant

### Knockdown of DLX6-AS1 leads to suppression of NB progression

**Fig. 2:**
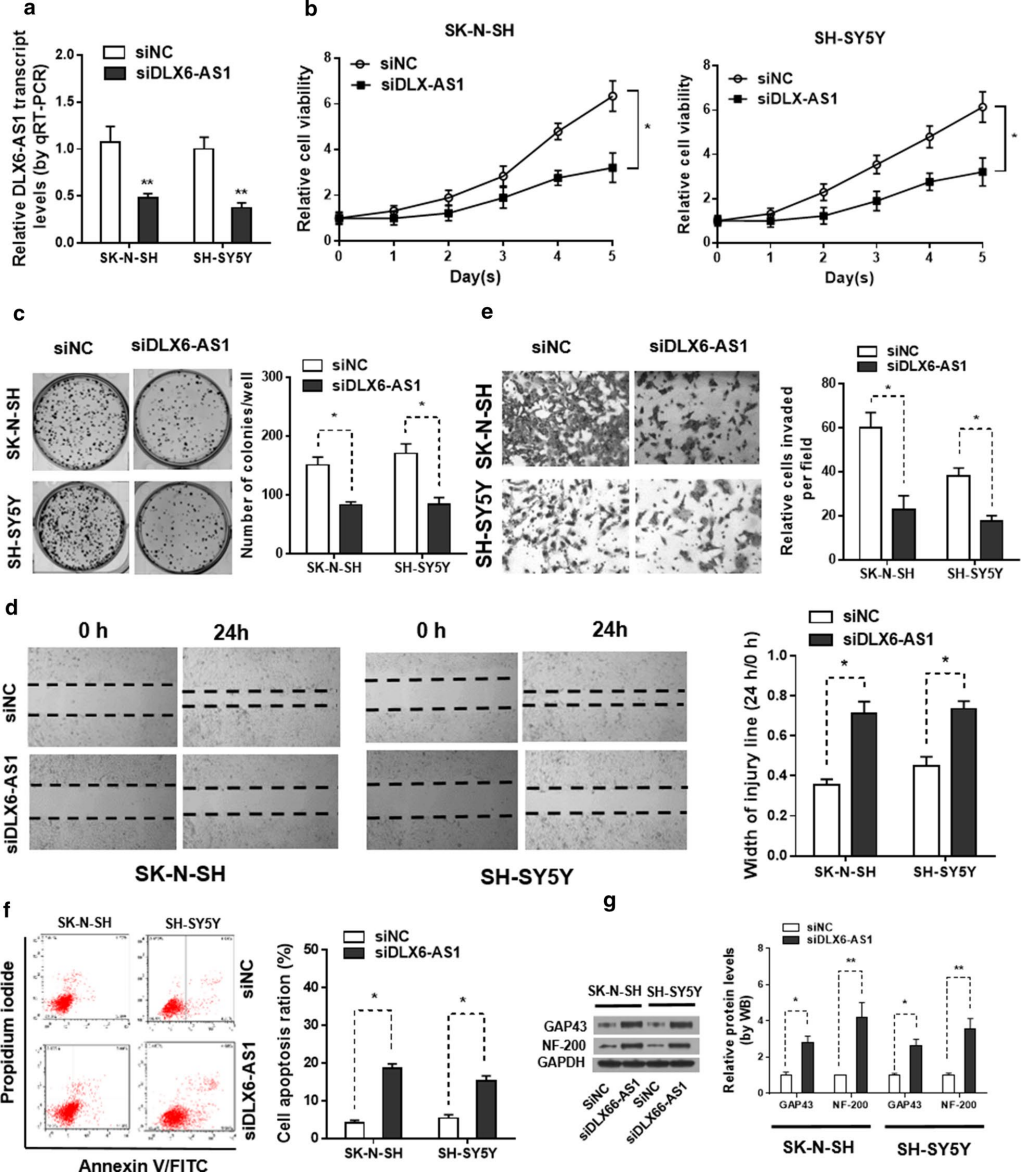
Downregulation of DLX6-AS1 inhibits NB cell proliferation, migration and invasion. **a** Relative expression levels of DLX6-AS1 in SK**‐**N**‐**SH and SH**‐**SY5Y cells after transfection with siNC or siDLX6-AS1 was examined by qRT**‐**PCR (24 h). Knockdown of siDLX6-AS1 markedly inhibited cell **b** viability and **c** proliferation in SK**‐**N**‐**SH and SH**‐**SY5Y cells detected by MTT assay (1–5 days) and colony formation assay (2 weeks). **d** Silenced DLX6-AS1 suppressed the cell migratory capacities of SK**‐**N**‐**SH and SH**‐**SY5Y cells as determined by a wound‐healing assay (24 h). **e** The results of the transwell assay (24 h) suggested that knockdown of DLX6-AS1 obviously inhibited the capacities of cell invasion in SK‐N‐SH and SH‐SY5Y cells. **f** Flow cytometry analysis (48 h) was conducted to analyze the apoptosis rates in SK**‐**N**‐**SH and SH**‐**SY5Y cells after transfection with siNC or siDLX6-AS1. **g** The protein levels of neuronal differentiation markers GAP43 and NF-200 were detected in SK**‐**N**‐**SH and SH**‐**SY5Y cells after transfection with siNC or siDLX6-AS1 by western blot (48 h). *p < 0.05 and **p < 0.01

As we revealed that DLX6-AS1 was significantly upregulated in NB, we then sought the mechanistic regulation of DLX6-AS1 in human NB development. To do so, we transfected SK-N-SH and SH-SY5Y cells with DLX6-AS1 siRNA (siDLX6-AS1) or scrambled siRNA (siNC). qPCR confirmed that, endogenous DLX6-AS1 genes were markedly downregulated in SK-N-SH and SH-SY5Y cells transfected with siDLX6-AS1 than in those transfected with siNC (Fig. 2a). For the proliferation capacity, MTT assay and colony formation assay together demonstrated that DLX6-AS1 knockdown inhibited the proliferation vitality of NB cells (Fig. 2b, c). Wound‐healing assays demonstrated that knockdown of DLX6-AS1 suppressed the migration ability of SK-N-SH and SH-SY5Y cells (Fig. 2d). Depletion of DLX6-AS1 led to decrease in invasion ability of SK-N-SH and SH-SY5Y cells in vitro (Fig. 2e). Furthermore, Annexin V/FITC and PI staining flow cytometry assay indicated that DLX6-AS1 knockdown induced the apoptosis of NB cells (Fig. 2f). Moreover, increased expression of neuronal differentiation markers growth associated protein 43(GAP43)and neurofilament heavy polypeptide (NF-200) were observed in SK-N-SH and SH-SY5Y cells following DLX6-AS1 suppression (Fig. 2g). Together, these findings demonstrated that knockdown of DLX6-AS1 suppressed the progression of NB.

### DLX6-AS1 regulates NB cell proliferation, migration, invasion and apoptosis by targeting miR‐107

**Fig. 3:**
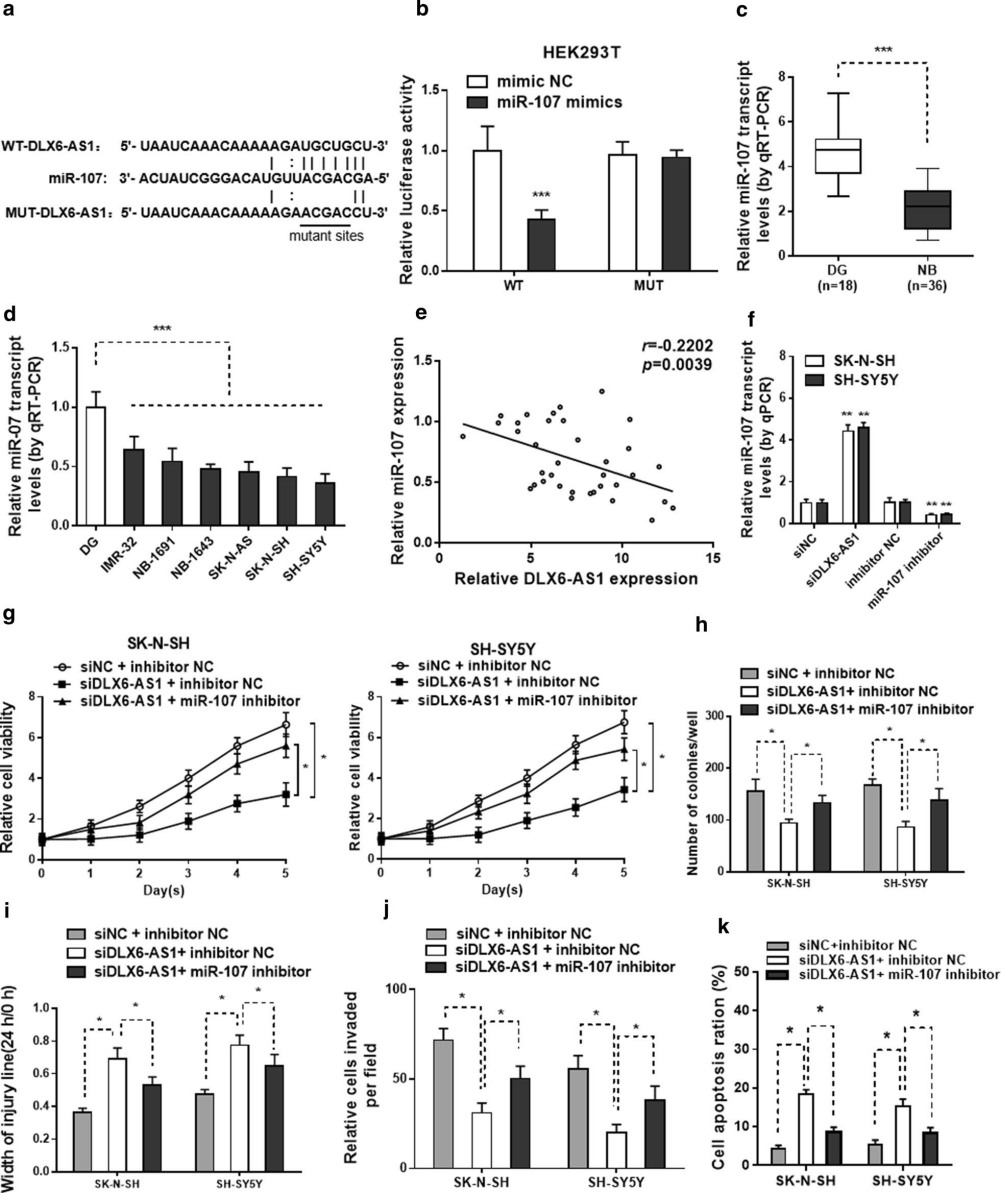
DLX6-AS1 can bind with miR-107 and knockdown of miR-107 attenuates siDLX6-AS1 inhibited effects on NB cell proliferation, migration and invasion. **a** The predicted binding sites of miR**‐**107 to the DLX6-AS1 sequence. **b** Luciferase reporter assays were performed for the detection of the luciferase activities of HEK**‐**293T cells after transfections. The qRT**‐**PCR results of miR**-**107 expression in **c** NB tissues and **d** NB cells. **e** Spearman’s correlation curve indicated a negative correlation between DLX6-AS1 and miR**‐**107 in NB tissues. **f** The relative miR**‐**107 expression in SK**‐**N**‐**SH and SH**‐**SY5Y cells after transfection with siNC, siDLX6-AS1, inhibitor NC or miR**‐**107 inhibitor was tested by qRT**‐**PCR analysis (24 h). **g** MTT assay (1–5 days) and **h** colony formation assay (2 weeks) results showed cell viability and cell proliferation abilities in two NB cells in different groups. Cell migration and invasion capacities in two NB cells with different transfections were determined by **i** wound healing assay (24 h) and **j** transwell assays (24 h), respectively. **k** Flow cytometry analysis (48 h) was conducted to analyze the apoptosis rates in SK**‐**N**‐**SH and SH**‐**SY5Y cells in different group. *p < 0.05, **p < 0.01 and ***p < 0.001

We identified that miR-107 as a potential target of DLX6-AS1 with Starbase (http://starbase.sysu.edu.cn). There is a putative binding sites of DLX6-AS1 and miR-107 (Fig. 3a). Luciferase reporter assays showed that miR-107 mimics significantly repressed the luciferase activity of which transfected with the reporter plasmid, which containing wild type DLX6-AS1 in the downstream of luciferase gene, but had no effect on the mutant one in HEK293T cells (Fig. 3b). Meanwhile, the miR-107 expression level in NB tissues and NB cells was lower than those in normal dorsal ganglia (Fig. 3c, d). Spearman’s correlation analysis revealed that miR‐107 expression was negatively correlated with DLX6-AS1 expression (Fig. 3e). qRT-PCR further validated that the silencing of DLX6-AS1 by siDLX6-AS1 increased the expression level of miR-107 in SK-N-SH and SH-SY5Y cell lines (Fig. 3f). The efficacy of miR-107 inhibitor or NC was also confirmed by qRT-PCR in these 2 NB cell lines. We observed miR-107 inhibitor significantly suppressed the expression of miR-107 when compared with cells transfected with NC (Fig. 3f).

Furthermore, to explore whether DLX6-AS1 promoted cell progression through miR-107, SK-N-SH and SH-SY5Y cells were treated with siDLX6-AS1, together with miR-107 inhibitor or NC. MTT assay showed that miR-107 inhibitor reversed cell viability as well as colony formation ability caused by DLX6-AS1 knockdown (Fig. 3g, h). In addition, cell migration and invasion assay showed that miR-107 inhibitor abrogated the inhibitory effect of DLX6-AS1 knockdown on cell migration and invasion in SK-N-SH and SH-SY5Y cells (Fig. 3i, j). Besides, knockdown of miR-107 abolished DLX6-AS1 depletion-induced apoptosis in NB cells (Fig. 3k). Taken together, these results supported that DLX6-AS1 promotes NB progression by targeting miR‐107 in NB.

### BDNF is a miR-107 target regulated by DLX6-AS1 and DLX6-AS1/miR-107/BDNF functions in NB progression

**Fig. 4:**
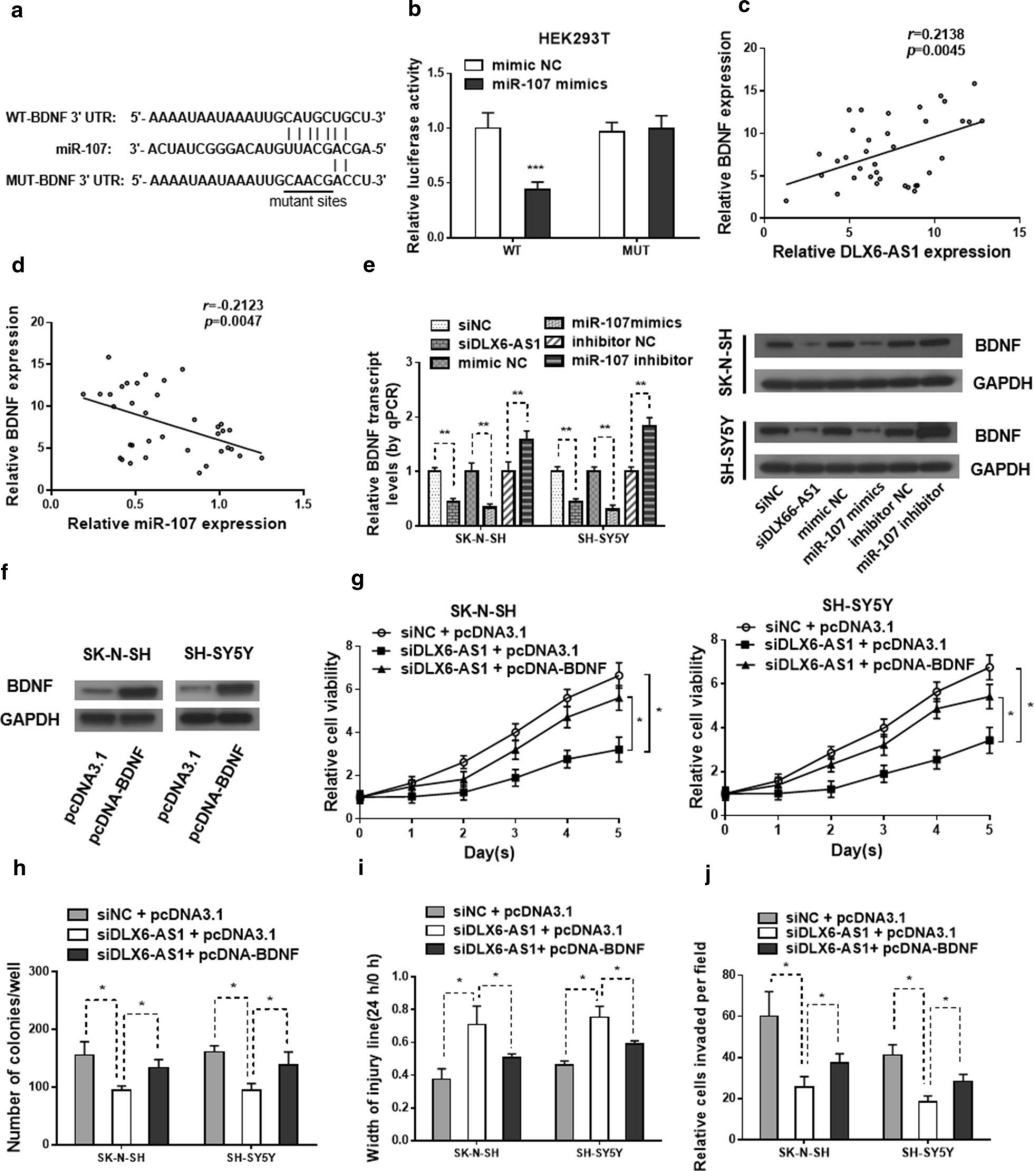
BDNF is the target of miR-107 regulated by DLX6-AS1 and the role of DLX6-AS1/miR**‐**107/BDNF axis in NB progression. **a** The predicted binding sites of BDNF to the miR**‐**107 sequence. **b** Luciferase reporter assay in HEK‐293T cells was used to confirm the binding between miR‐107 and BDNF. Spearman’s correlation analysis uncovered the association between BDNF expression and **c** miR**‐**107 or **d** DLX6-AS1 level in NB tissues. **e** Relative BDNF expression in SK**‐**N**‐**SH and SH**‐**SY5Y cells transfected with siNC, siDLX6-AS1, mimic NC, miR-107 mimics, inhibitor NC or miR**‐**107 inhibitor was tested by qRT**‐**PCR (24 h) and western blot (48 h). **f** Western blot analysis (48 h) was applied to evaluate the protein level of BDNF expression in pcDNA3.1 or pcDNA-BDNF transfected SK‐N‐SH and SH‐SY5Y cells. **g** MTT assay (1–5 days) and **h** colony formation assay (2 weeks) results showed cell viability and cell proliferation abilities in two NB cells in different group. Cell migration and invasion capacities in two NB cells with different transfections were determined by **i** wound healing assay (24 h) and **j** transwell assays (24 h), respectively. *p < 0.05, **p < 0.01 and ***p < 0.001

Bioinformatic analysis indicated that BDNF contains a miR-107 binding site in its 3′UTR (Fig. 4a). MiR-107 significantly reduced the luciferase activity in HEK293T cells transfected with the wild-type BDNF plasmid, but it failed to suppress luciferase activity in cells transfected with the mutant plasmid (Fig. 4b). The mRNA expression level of BDNF was upregulated in NB cases (GSE16476) with the status of death and in our collected 36 NB patient tissues compared with normal dorsal ganglia (Additional file 1: Figure S1a, b). As compared with normal dorsal ganglia, higher protein level of BDNF was also seen in all NB cell lines (Additional file 1: Figure S1c). The Spearman’s correlation analysis suggested that BDNF expression was negatively correlated with miR‐107 level but positively correlated with DLX6-AS1 expression in NB tissues (Fig. 4c, d). The mRNA and protein levels of BDNF was markedly reduced in SK-N-SH and SH-SY5Y cells transfected with the miR-107 mimics or siDLX6-AS1, while increased by transfected with miR-107 inhibitor (Fig. 4e and Additional file 1: Figure S1d). Then the effect of BDNF over-expression plasmid on SK-N-SH and SH-SY5Y cells was confirmed by western blot (Fig. 4f and Additional file 1: Figure S1e). Interestingly, restoration of BDNF rescued the inhibitory effect on cell proliferation, migration, and invasion in the SK-N-SH and SH-SY5Y cells induced by knockdown of DLX6-AS1 (Fig. 4g–j). Besides, overexpression of BDNF prominently abated DLX6-AS1 depletion-induced apoptosis in NB cells (Additional file 1: Figure S1f). These data illustrated that DLX6-AS1 functions in NB progression through modulating the miR‐107/BDNF pathway.

### Inhibition of DLX6-AS1 suppressed tumor growth in vivo

**Fig. 5:**
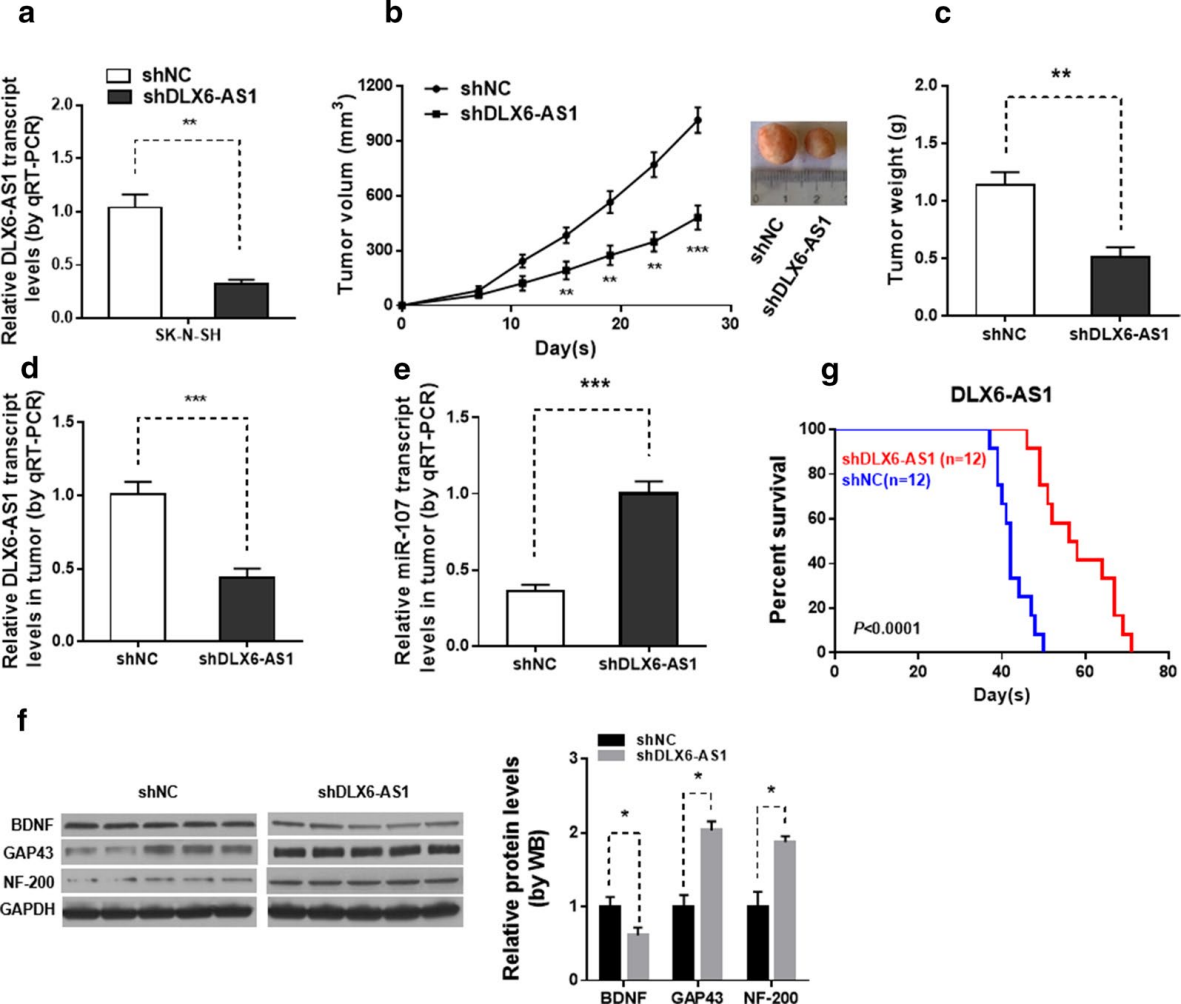
Knockdown of DLX6-AS1 inhibits tumor growth in vivo. **a** Relative expression levels of DLX6-AS1 in SK**‐**N**‐**SH cells stably transfected with shNC or DLX6-AS1 by qRT**‐**PCR. **b** Tumor volume was measured in shNC group and shDLX6-AS1 group at indicated times. **c** The weight of dissected tumors in shDLX6-AS1 group was lower than that in shNC group. The expression of **d** DLX6-AS1 and **e** miR-107 in dissected tumor tissues from shNC group and shDLX6-AS1 group were determined by qRT-PCR. **f** The BDNF, GAP43 and NF-200 protein expression of dissected tumor tissues were determined by western blot. **g** Survival analysis showing the overall survival of mice treated with shNC or shDLX6-AS1 SH-SY5Y cells. **p < 0.01 and ***p < 0.001

To investigate whether knockdown of DLX6-AS1 could inhibit tumor growth in vivo, SK-N-SH cells stably infected with sh‐DLX6-AS1 or sh‐NC were injected subcutaneously into nude mice. qPCR confirmed that, endogenous DLX6-AS1 genes were markedly downregulated in SK-N-SH cells transfected with shDLX6-AS1 than in those transfected with shNC (Fig. 5a). Tumor volumes were measured every 4 days until the mice were killed at 28 days. Compared with sh‐NC cell‐derived xenograft tumors, sh‐DLX6-AS1 cell‐derived xenograft tumors grew more slowly (Fig. 5b). Moreover, the mean weight of sh‐DLX6-AS1 cell‐derived xenograft tumors were also significantly decreased compared with sh‐NC cell‐derived xenograft tumors (Fig. 5c). The qRT‐PCR assay revealed that DLX6-AS1 expression was downregulated, while miR‐107 expression was upregulated in sh‐DLX6-AS1 cell‐derived xenograft tumors compared with sh‐NC cell‐derived xenograft tumors (Fig. 5d, e). In addition, the protein level of BDNF was decreased while GAP43 and NF-200 was upregulated in shDLX6-AS1 group (Fig. 5f). Moreover, mice injected with sh‐DLX6-AS1 cells had a longer survival time than their controls (Fig. 5f).

## Discussion

LncRNAs, generally defined as RNA polymerase II transcripts that are longer than 200 nucleotides but lack significant protein-coding capacity [14, 15], are important regulators of gene transcription, tumor initiation and progression [16]. However, the lncRNAs correlated with the tumorigenesis and development of NB still remain largely unknown. In this study, we discovered that DLX6-AS1 was significantly upregulated in NB tissues and cell lines. Down-regulation of DLX6-AS1 promoted neuronal differentiation and exerted suppressive effects on NB cell proliferation, migration, and invasion while induced apoptosis in NB cells which provides new evidence that DLX6-AS1 might act as an oncogenic role in NB tumorigenesis and development.

The regulation mechanism between lncRNAs and miRNAs are complicated [17]. Increasing studies have proposed a ceRNA hypothesis, by which lncRNAs act as miRNA sponges to suppress the expressions and biological functions of miRNAs, and thereby depress miRNA function [18]. For example, lncRNA 1308 function as a competing endogenous RNA (ceRNA) for miR-124 to regulate cell invasion through the miR-124/ADAM 15 signaling pathway in non-small-cell cancer [19]. DLX6-AS1 promoted pancreatic cancer cell proliferation and invasion by attenuating the endogenous function of miR-181b [20]. In the present study, we notified that DLX6-AS1 work as a ceRNA to sponge miR-107 in NB cells. MicroRNA-107 has been reported to regulate several types of tumorigenesis and progression. For example, miR-107 inhibits Head and neck squamous cell carcinoma cell proliferation and invasion through suppression of PKCɛ [21]. Depletion of the tumor suppressor miR-107 in plasma relates to pancreatic cancer progression and poor outcomes [22]. However, in contradiction to the above studies, Chen et al. revealed that miR-107 expression increased the tumorigenic and metastatic potential of a human breast cancer cell line in mice via inhibition of let-7 and upregulation of let-7 targets [23]. Taken together, these results indicate that the biology of miR-107 is complex and highly cell-type dependent. In this study, we uncovered that miR-107 acted as a tumor suppressor in NB. Knockdown of miR-107 reversed the function of cell viability, migration, invasion and apoptosis abilities caused by DLX6-AS1 depletion.

Bioinformatics analysis revealed that BDNF was the potent target of miR-107. BDNF, located on the short arm of chromosome 11 (11p13), is up-regulated in various human cancer types including NB [24]. Several studies indicate that BDNF increases NB cell survival [25] and cell invasion [26], and protects cells from chemotherapy [27]. In this study, we found that DLX6-AS1 expression was negatively correlated with miR‐107 expression, and positively correlated with BDNF expression both in NB cells and NB tissues, respectively. We further demonstrated that restoration of BDNF reversed the effects of DLX6-AS1 knockdown on cell proliferation, migration, invasion and apoptosis in NB cells. In vivo assays showed that DLX6-AS1 and BDNF expression were downregulated, while miR‐107, GAP43 and NF-200 expression was upregulated in sh‐DLX6-AS1 cell‐derived xenograft tumors compared with sh‐NC cell‐derived xenograft tumors. Taken together, we can conclude that DLX6-AS1 exerts an oncogenic activity in NB by regulating the miR‐107/BDNF pathway.

## Conclusion

In summary, we demonstrated that DLX6-AS1 was upregulated in NB tissues and cell lines. In addition, we discovered that the DLX6-AS1 suppressed neuronal differentiation and apoptosis while promoted cell proliferation, migration, and invasion through the miR‐107/BDNF pathway. Thus, our study provides further insight into the molecular mechanism of lncRNAs in NB tumorigenesis, which may promote the development of lncRNA-directed diagnosis and therapy for NB.

## Supplementary information

**Additional file 1: Figure S1.** Effects of DLX6-AS1 on the expression of BDNF in NB and on the apoptosis in NB cells. **a** Mining of the microarray dataset (GSE16476) revealed the BDNF transcript level in NB tissues with the status of death. **b** qRT**‐**PCR assay indicated the BDNF transcript level in NB tissues (n = 36) compared with normal dorsal ganglia (DG, pooling n = 18). **c** Western blot assays showed the protein level of BDNF in normal dorsal ganglia (DG) and cultured NB cell lines. The protein level of BDNF in SK**‐**N**‐**SH and SH**‐**SY5Y cells after transfection with d siNC, siDLX6-AS1, mimic NC, miR-107 mimics, inhibitor NC or miR**‐**107 inhibitor and e pcDNA3.1 or pcDNA-BDNF plasmid was examined by western blot (48 h). **f** Flow cytometry analysis (48 h) was conducted to analyze the apoptosis rates in SK**‐**N**‐**SH and SH**‐**SY5Y cells in different group. *p < 0.05 and **p < 0.01.

## Data Availability

All data generated or analysed during this study are included in this published article.

## Abbreviations

NB: neuroblastoma
DLX6-AS1: DLX6 antisense RNA 1
BDNF: brain derived neurotrophic factor
shRNA: small hairpin RNA
FBS: fetal bovine serum
