# Supplementary material for: Long noncoding RNA DLX6-AS1 promotes neuroblastoma progression by regulating miR-107/BDNF pathway
Source: Cancer Cell Int. 2019 Nov 27;19:313. doi: 10.1186/s12935-019-0968-x (PMC6880520; doi:10.1186/s12935-019-0968-x)
Supplement: Supplementary file 1 — Additional file 1: Figure S1. Effects of DLX6-AS1 on the expression of BDNF in NB and on the apoptosis in NB cells. a Mining of the microarray dataset (GSE16476) revealed the BDNF transcript level in NB tissues with the status of death. b qRT‐PCR assay indicated the BDNF transcript level in NB tissues (n = 36) compared with normal dorsal ganglia (DG, pooling n = 18). c Western blot assays showed the protein level of BDNF in normal dorsal ganglia (DG) and cultured NB cell lines. The protein level of BDNF in SK‐N‐SH and SH‐SY5Y cells after transfection with d siNC, siDLX6-AS1, mimic NC, miR-107 mimics, inhibitor NC or miR‐107 inhibitor and e pcDNA3.1 or pcDNA-BDNF plasmid was examined by western blot (48 h). f Flow cytometry analysis (48 h) was conducted to analyze the apoptosis rates in SK‐N‐SH and SH‐SY5Y cells in different group. *p < 0.05 and **p < 0.01. [file 12935_2019_968_MOESM1_ESM.docx]

Figure S1


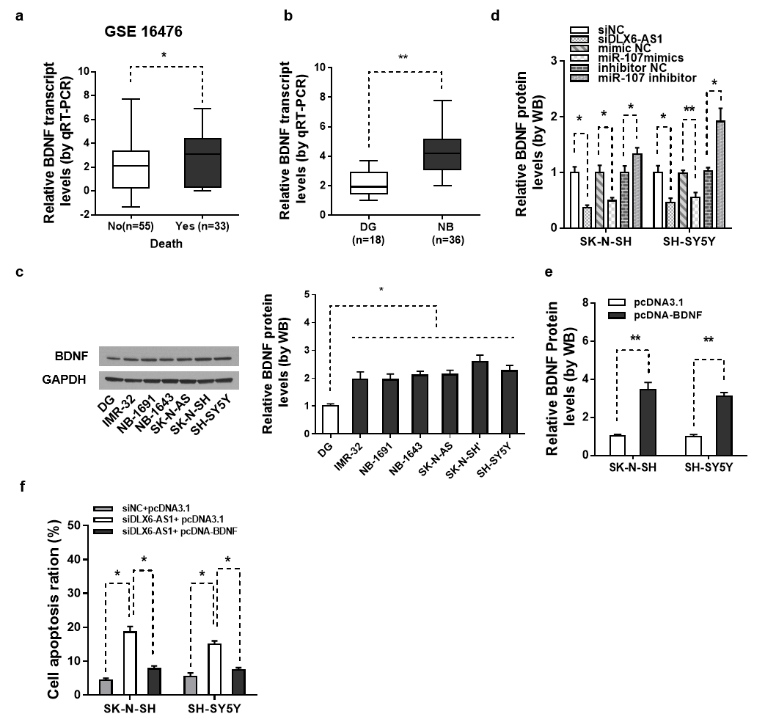


Figure S1 Effects of DLX6-AS1 on the expression of BDNF in NB and on the apoptosis in NB cells. **a** Mining of the microarray dataset (GSE16476) revealed the BDNF transcript level in NB tissues with the status of death. b qRT**‐**PCR assay indicated the BDNF transcript level in NB tissues (n=36) compared with normal dorsal ganglia (DG, pooling n=18). **c** Western blot assays showed the protein level of BDNF in normal dorsal ganglia (DG) and cultured NB cell lines. The protein level of BDNF in SK**‐**N**‐**SH and SH**‐**SY5Y cells after transfection with d siNC, siDLX6-AS1, mimic NC, miR-107 mimics, inhibitor NC or miR**‐**107 inhibitor and e pcDNA3.1 or pcDNA-BDNF plasmid was examined by western blot (48hr). **f** Flow cytometry analysis (48hr) was conducted to analyze the apoptosis rates in SK**‐**N**‐**SH and SH**‐**SY5Y cells in different group**.** **p* < 0.05 and ***p* < 0.01
